# Sequencing and de novo assembly of the Koshihikari genome and identification of the genomic region related to the eating quality of cooked rice

**DOI:** 10.1007/s11032-022-01335-3

**Published:** 2022-10-14

**Authors:** Yoon Kyung Lee, Yunjoo Lee, Su Jang, Taeyoung Lee, Mi-Ok Woo, Jeonghwan Seo, Backki Kim, Hee-Jong Koh

**Affiliations:** 1 Department of Agriculture, Forestry and Bioresources, Plant Genomics and Breeding Institute, Research Institute for Agriculture and Life Sciences, Seoul National University, Seoul, South Korea; 2 Bioinformatics Institute, Macrogen Inc, Seoul, 08511 Republic of Korea; 3 Science & Technology Policy Division, Ministry of Agriculture, Food and Rural Affairs, Sejong, South Korea; 4 Crop Breeding Division, National Institute of Crop Science, Rural Development Administration, Wanju, 55365 Korea

**Keywords:** De novo genome assembly, *Oryza sativa*, *Japonica*, Koshihikari, Nanopore sequencing, Eating quality

## Abstract

The *japonica* rice (*Oryza sativa* L.) cultivar Koshihikari is considered an important breeding material with good eating quality (EQ). To effectively utilize Koshihikari in molecular breeding programs, determining its whole genome sequence including cultivar-specific segment is crucial. Here, the Koshihikari genome was sequenced using Nanopore and Illumina platforms, and de novo assembly was performed. A highly contiguous Koshihikari genome sequence was compared with Nipponbare, the reference genome of *japonica*. Genome-wide synteny was observed, as expected, without large structural variations. However, several gaps in alignment were detected on chromosomes 3, 4, 9, and 11. It was notable that previously identified EQ-related QTLs were found in these gaps. Moreover, sequence variations were identified in chromosome 11 at a region flanking the P5 marker, one of the significant markers of good EQ. The Koshihikari-specific P5 region was found to be transmitted through the lineage. High EQ cultivars derived from Koshihikari possessed P5 sequences; on the other hand, Koshihikari-derived low EQ cultivars didn’t contain the P5 region, which implies that the P5 genomic region affects the EQ of Koshihikari progenies. The EQ of near-isogenic lines (NILs) of Samnam (a low EQ cultivar) genetic background harboring the P5 segment was improved compared to that of Samnam in Toyo taste value. The structure of the Koshihikari-specific P5 genomic region associated with good EQ was analyzed, which is expected to facilitate the molecular breeding of rice cultivars with superior EQ.

**Supplementary Information:**

The online version contains supplementary material available at 10.1007/s11032-022-01335-3.

## Introduction

Rice (*Oryza sativa* L.) is the staple food of 3.5 billion people worldwide and has been extensively studied as a model crop in genomics research. *O. sativa* is divided into two subspecies, *japonica* and *indica* (Izawa and Shimamoto 1996). According to a recent within-species diversity analysis, *O. sativa* could be further divided into nine subpopulations, based on genetic variation and population structure (Wang et al. 2018). Advances in sequencing technologies have enabled the identification of different kinds of sequence variations, thus unraveling the genomic diversity within a given species. The 3000 Rice Genomes Project was established to comprehend the genomic diversity within *O. sativa*. The genomes of 3024 rice accessions were sequenced using the Illumina platform by preparing 500-bp-insert DNA libraries, generating 17 × 10^9^ bp of data (Li et al. 2014). Numerous single-nucleotide polymorphisms (SNPs) and other forms of variations such as structural variations and gene presence/absence variations were identified when aligned with the Nipponbare reference genome sequence (Hu et al. 2018).

Koshihikari is a *japonica* rice cultivar developed in 1956 in Japan. With its superior agronomic characteristics such as adaptability to diverse environments, tolerance to pre-harvest sprouting, cold tolerance during booting stage, and most importantly good EQ and stickiness of cooked rice, Koshihikari is one of the most widely cultivated rice cultivars in Japan (Kobayashi et al. 2018). Because of its popularity in the market, the unique agronomic features of Koshihikari have been studied extensively. Ohtsubo et al. (2002) developed molecular markers including P5, B43, and M11 to differentiate Koshihikari and Koshihikari-derived cultivars from other rice genotypes and to utilize the former cultivars for molecular breeding purposes. Several major-effect quantitative trait loci (QTLs) underlying important traits such as heading date (Matsubara et al. 2012), grain quality (Takeuchi et al. 2008), and other physiological characteristics (Hori et al. 2010) have been identified using segregating populations, the generation of which is time-consuming and labor-intensive. In an attempt to better understand the unique superior characteristics of Koshihikari, its whole genome sequencing was conducted using next-generation sequencing (NGS) techniques, and their genome composition was studied using SNP information. Although approximately 67,000 SNPs were discovered between Koshihikari and Nipponbare, the draft genome sequence of Koshihikari was highly fragmented with thousands of scaffolds (Yamamoto et al. 2010). Therefore, there still is limitation in explaining the cultivar-specific characteristics only with the haplotypes by means of genome-wide SNPs from fragmented scaffolds.

Of the several criteria used to determine the quality of rice, EQ is the most important trait. Physicochemical properties determine the cooking and EQ of rice, and granule-bound starch synthase (Wang et al. 1995) and starch synthase II (Gao et al. 2011) are mainly responsible for these properties. Several studies have attempted to identify the genetic regions related to the good EQ of Koshihikari. For example, two QTLs associated with the stickiness of cooked rice were identified using the double-haploid lines of Akihikari and Koshihikari (Takeuchi et al. 2007); 21 QTLs associated with the EQ of rice were discovered using the Koshihikari/Kasalath//Koshihikari backcross inbred lines and chromosome segment substitution lines (CSSLs) (Ebitani et al. 2005); and 43 QTLs responsible for various physicochemical properties were detected in the recombinant inbred lines (RILs) derived from Moritawase and Koshihikari (Wada et al. 2008). Additionally, molecular markers strongly associated with the EQ of rice were identified (Lestari et al. 2009), and one marker, P5, was specifically detected from Koshihikari and its related cultivar Hitomebore.

Advances in scientific knowledge and the related technologies lead to limitless possibilities in understanding the genetic basis of agronomic traits. The genome sequence of *O. sativa* was the first to be assembled using the Sanger sequencing technique (International Rice Genome Sequencing Project and Sasaki 2005; Michael and VanBuren 2015). Completion of the Nipponbare reference genome sequence enabled the resequencing of important rice cultivars, which in turn led to the identification of SNPs and short insertion/deletion mutations (InDels) associated with important agricultural traits. However, resequencing is not applicable to highly diversified regions. Recently, long-read sequencing has been used to obtain chromosome-level genome sequences of important cultivars. The whole genome sequence of IR64 was determined using linked-read and Nanopore sequencing approaches. The de novo genome assembly technique produced a highly contiguous genome of IR64, with an estimated size of 367 Mb (Tanaka et al. 2020). Additionally, high-quality reference genomes of Basmati 334 and Dom Sufid were successfully generated using Nanopore sequencing (Choi et al. 2020). The assembled genomes were highly contiguous, and structural variations and presence/absence variations were well characterized.

In this study, we constructed a high-quality de novo assembly of the Koshihikari genome using both Nanopore long-read and Illumina short-read sequencing. Furthermore, cultivar-specific genomic regions associated with the good EQ of Koshihikari were identified from the assembled genome sequences of Koshihikari and were verified using NILs.

## Results

### De novo assembly of Koshihikari genome using Nanopore and Illumina sequencing reads

The Koshihikari genome was sequenced using the Oxford Nanopore Technologies GridION platform and Illumina MiSeq platform. Long-read sequencing generated 3,510,702 reads (~ 16 Gb), while short-read paired-end resequencing generated 34,940,048 reads (18.4 Gb) (Table 1).

**Table 1:** Statistics of sequencing data

	ONT GridION	Illumina MiSeq
Number of reads	3,510,702	34,940,048
Total bases (bp)	15,985,579,646	18,418,445,998
Sequencing depth	42 ×	49 ×

To obtain a high-quality genome sequence, long and short reads were corrected prior to the initial assembly and used in polishing the draft genome. Since Koshihikari is a *japonica* cultivar and share similarities with Nipponbare in their whole genomes, a reference-guided scaffolding was adopted. Consequently, a 348.7-Mb genome assembly of Koshihikari, with 1530 contigs and 161 scaffolds, was obtained (Table 2). Numerous contigs, ranging from 228 bp to 123 kb in size, remained unscaffolded. The Benchmarking Universal Single-Copy Orthologs (BUSCO) gene completion of assembly was 98.5% of Embryophyta gene groups, which is similar to that of Nipponbare (98.4%) (International Rice Genome Sequencing Project and Sasaki 2005).

**Table 2:** Summary of the Koshihikari genome assembly

	Koshihikari
Number of contigs	1530
Number of scaffolds	161
Total number of bases in contigs	348,716,585
Total number of scaffolded bases	348,853,485
Contig N50 length	536.46 kb
Contig L50	178
Scaffold N50 length	27.74 Mb
Scaffold L50	6
Maximum contig length	2.95 Mb
Maximum scaffold length	41.47 Mb
GC content	43.24%
BUSCO gene completion	98.5%

Based on the high-quality Koshihikari draft genome sequence, protein-coding genes were predicted using the MAKER program (version 2.31.11) (Cantarel et al. 2008) and a dataset publicly available at the Rice Annotation Project Database (RAP-DB) (Sakai et al. 2013). A total of 46,275 genes were annotated, and the BUSCO gene completion of gene annotation was 89.4% of 1614 total gene groups from the Embryophyta dataset.

### Genome comparison and structure analysis

**Fig. 1:**
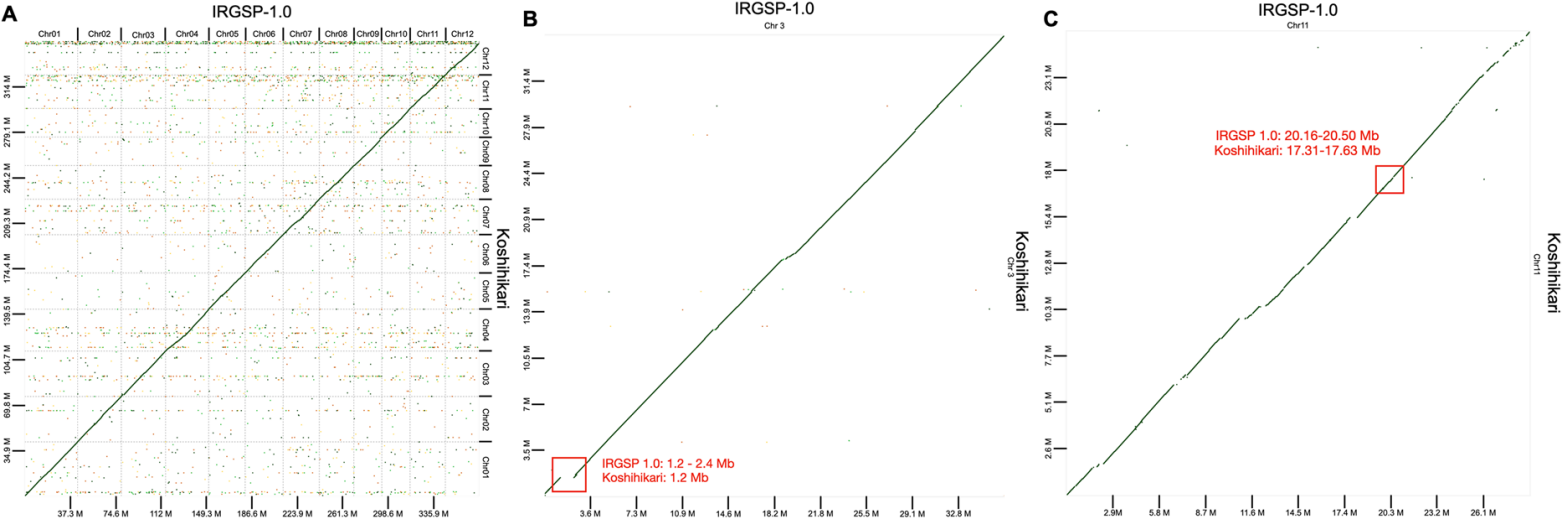
Alignment of the Koshihikari draft genome and Nipponbare reference genome sequence. **A** Alignment of all chromosomes, **B** chromosome 3, and **C** chromosome 11. Alignment blocks with greater than 90% sequence identity are shown. Red box indicates the candidate region

Alignment of the Koshihikari draft genome with the Nipponbare reference genome revealed a highly conserved genome structure (Fig. 1A). No large structural variations were detected between the two genomes, although gaps in alignment were observed on chromosomes 3, 4, 9, and 11. Among these gaps, QTLs in EQ and stickiness, qOE3, were previously reported in chromosome 3 in Fig. 1B (Takeuchi et al. 2008), and Koshihikari-specific molecular marker associated with EQ, P5, was reported in chromosome 11 (Lestari et al. 2009). Additionally, several regions of low similarity between Nipponbare and Koshihikari genomes were identified in chromosome 11 (Fig. 1C).

**Fig. 2:**
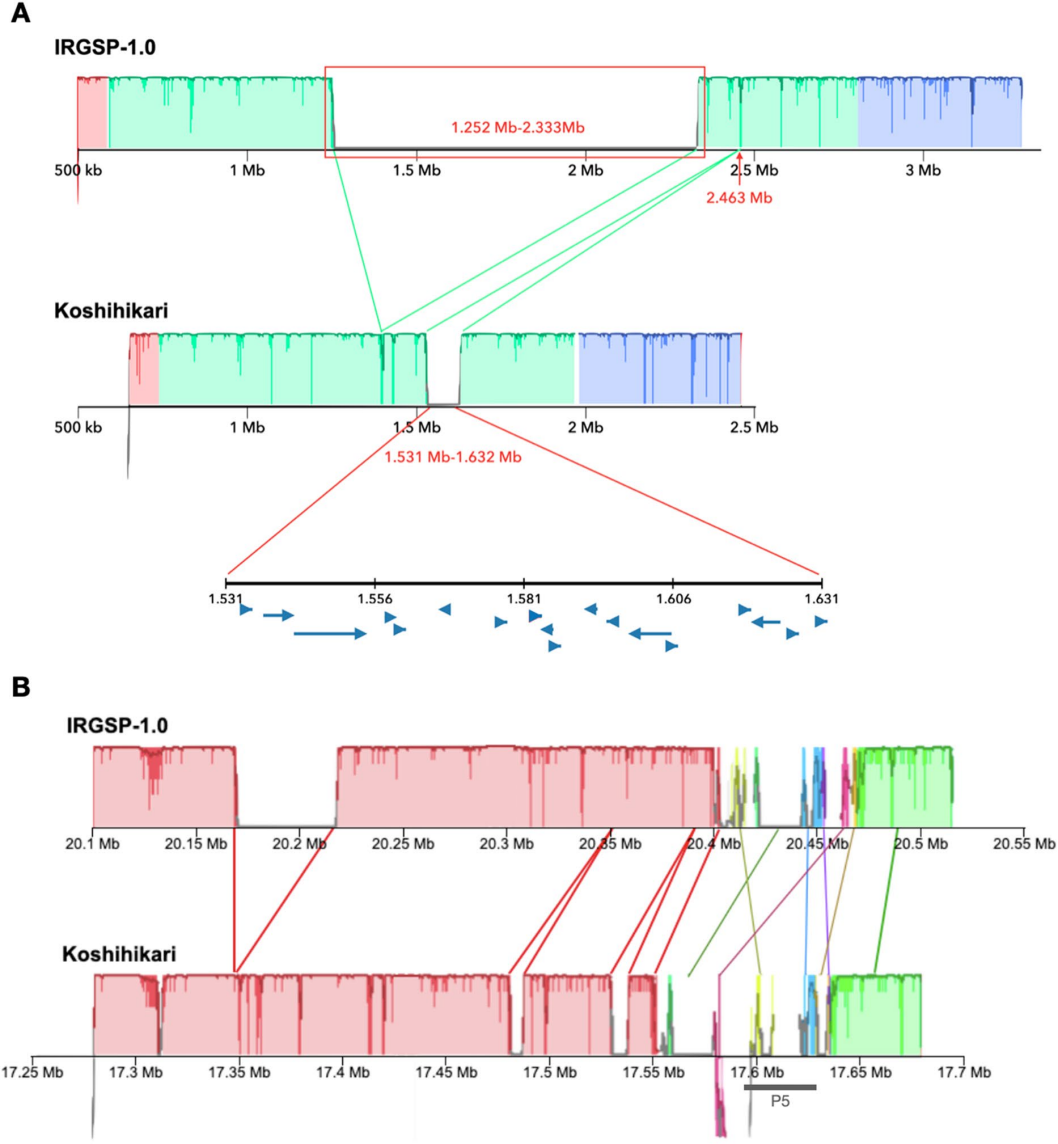
Structure analysis of gapped regions. **A** Region nearby qOE3 in chromosome 3. **B** Region flanking P5 marker in chromosome 11. Genomic regions showing significant differences in structure are compared. Lines with different colors indicate regions containing deletions, insertions, and inversions. Blue arrows indicate the predicted genes in the region. Gray line labeled with P5 indicates the position of P5 marker in the genome

A structure of gapped region in qOE3 was depicted in detail by comparison of Koshihikari and Nipponbare sequences using Mauve (Fig. 2A), and the sequence differences were validated using PCR analysis (Additional file 3: Fig. S2). By comparing the structures, the physical position of 1.25 Mb to 2.33 Mb of Nipponbare sequence was deleted in Koshihikari draft genome. In this considerable size of deleted sequence, 172 annotated genes based on RAP-DB were included (Additional file 1: Table S2). Notably, genes like Os03g0128100, 1,3-beta-glucan synthase component family protein; Os03g0129300, beta subunit of glyceraldehyde-3-phosphate dehydrogenase; and Os03g0141200, beta-amylase PCT-BMYI, were part of the deleted region. Moreover, there was about 100 kb of Koshihikari-specific insertion sequence based on Nipponbare sequence at 2.46 Mb. The gene prediction analysis of Koshihikari draft genome revealed that 18 genes were predicted in the 100 kb of Koshihikari insertion sequence which functions include glucosaminyl(N-acetyl) transferase (Additional file 1: Table S3).

The sequence of P5 marker was searched using BLAST and aligned to one of the gaps on chromosome 11 of Koshihikari. The detailed structure of P5 region was explored to determine the sequence variation within the region. The Koshihikari draft genome sequence contained several deletions, insertions, and inversions compared with the Nipponbare reference (Fig. 2B) and confirmed with PCR analysis (Additional file 3: Fig. S2). A 17.55–17.64-Mb region on chromosome 11 of Koshihikari, which contained the P5 marker, showed low sequence similarity with the corresponding region in the Nipponbare genome. PCR was previously performed using P5 markers to Koshihikari-related cultivars, and Norin 1, one of the parental cultivars of Koshihikari, also had P5 segment indicating that P5 marker region of Koshihikari was derived from Norin 1. This region was also detected from other Koshihikari-derived improved cultivars such as Akitakomachi, Kinuhikari, Yumehikari, and Itadaki (data not shown).

**Fig. 3:**
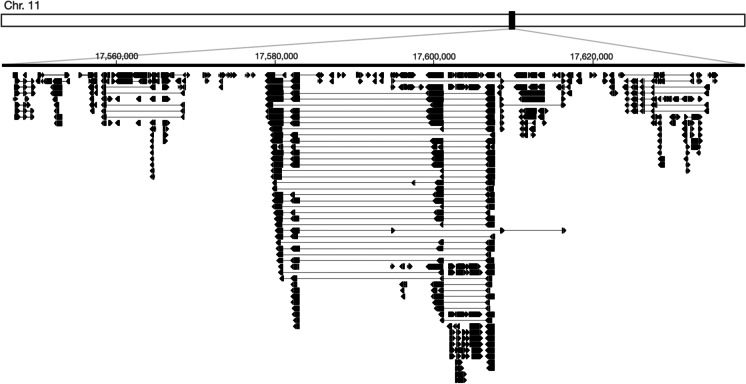
Prediction of genes in the novel Koshihikari-specific sequences in chromosome 11. Visualization of the predicted gene forms within the candidate region in the Koshihikari draft genome is shown

The genetic diversity of the novel Koshihikari-specific sequences could contribute to phenotypic differences associated with the EQ of cooked rice. Gene prediction analysis revealed that this 17.55–17.64-Mb region harbored numerous protein-coding genes (Fig. 3), some of which were present in the Nipponbare genome, while others were novel. The function of these genes was annotated as mostly hypothetical or unknown in rice. However, a couple of the predicted genes were annotated as related to the cell wall proteins and polysaccharides, such as glycine-rich cell wall structural protein, peptidase A1 domain-containing protein, and glycosyl transferase family protein (Additional file 1: Table S1).

### Read depth analysis

**Fig. 4:**
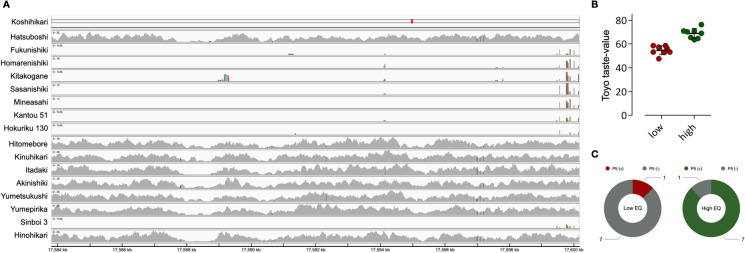
Comparison of EQ and presence/absence of P5 sequence in Koshihikari-derived cultivars. **A** Sequencing read alignment depth of Koshihikari-derived cultivars in P5 marker region. **B** Toyo taste values of EQ groups. **C** Comparison of presence/absence based on EQ groups

The P5 marker was previously reported as selection marker distinguishing Koshihikari (Ohtsubo et al. 2002) and also had been used in regression model to predict EQ with its high power of explanation (Lestari et al. 2009). The region was, therefore, further studied in detail for its association with EQ. With the knowledge of the presence of cultivar-specific sequences that possibly affect its good EQ trait in Koshihikari, the whole genome sequencing read depths of Koshihikari-derived cultivars in the P5 region were demonstrated and compared with the Toyo taste values of the cultivars (Fig. 4 and Additional file 4: Fig. S3). The improved cultivars which Koshihikari was used as breeding material in generation of the variety were selected and divided into two groups based on the EQ. The Toyo taste value of low EQ group was ranged from 47 to 58, 54.8 in average, and that of high EQ group was from 64 to 76, 68.9 in average (Fig. 4B. Out of 8 low EQ cultivars, only Hatsuboshi was aligned to Koshihikari draft genome in P5 marker region. On the other hand, 7 out of 8 high EQ cultivars were aligned to Koshihikari draft genome, except for Sinboi 3 (Fig. 4A, C). The results clearly indicated that the presence of P5 sequence in the genome could lead to the improvement in EQ.

### Association of the P5 region with eating quality

To validate the association of the Koshihikari-specific sequence of the P5 marker region with its good EQ, NILs of Samnam (a low EQ cultivar) harboring the Koshihikari-specific P5 region were developed (Additional file 2: Fig. S1). NILs that show the highest homogeneity to Samnam, 95.8%, 96.8%, and 96.9% of recurrent parent genome recovery (Additional file 1: Table S5), respectively, were selected, and several traits related to the EQ of NILs were compared with that of the parental cultivars (Table 3). The differences between Samnam and Koshihkari were noticeable in most of the traits except for the amylose content and breakdown viscosity. NILs did not show significant differences compared to that of Samnam in most of the traits. However, the Toyo taste values of NILs were notably higher than Samnam, ranged from 51.7 to 57.7, which indicate that the EQ of NILs was generally improved by Koshihikari-specific P5 segments.

**Table 3:** The phenotypic comparison of NILs and parent cultivars

	Samnam	Koshihikari	NIL-1	NIL-2	NIL-3
Amylose content (%)	16.6^a^	17.05^a^	16.55^a^	17.45^a^	17.0^a^
Protein content (%)	7.8^a^	6.55^b^	7.5^a^	7.6^a^	7.5^a^
Maximum viscosity (RVU)	250.6^b^	290.1^a^	248.9^b^	242.3^b^	241.8^b^
Minimum viscosity (RVU)	149.4^c^	176.8^a^	143.9^c^	149.2^c^	154.1^b^
Final viscosity (RVU)	251.2^b^	274.7^a^	247.0^c^	257.9^b^	255.6^b^
Breakdown viscosity (RVU)	101.1^a^	113.4^a^	104.9^a^	93.1^b^	87.7^c^
Gelatinization temperature (℃)	84.4^b^	82.1^a^	84.1^b^	85.2^b^	85.2^b^
Toyo taste value	50.7^c^	65.3^a^	51.7^c^	55.3^b^	57.7^b^

The different superscripts indicate significant differences of NILs and parental cultivars at *p* < 0.05 on each phenotype by Tukey's HSD test

## Discussion

With the increasing popularity of long-read sequencing in genomics studies, the related pipelines and methods have been developed (Pennisi 2017; Huddleston et al. 2014; Mahmoud et al. 2019; Amarasinghe et al. 2020). The unresolved genomic regions of Koshihikari were analyzed using a long-read and short-read hybrid approach. With a moderate sequencing depth, sequence correction using Illumina short reads, and reference-guided hybrid assembly, a significantly improved, high-quality Koshihikari draft genome sequence was assembled. The draft genome of Koshihikari assembled in this study showed high contiguity. However, generating a satisfactory genome assembly requires the use of additional technologies such as Bionano optical mapping and Hi-C sequencing, especially for more complex genomes (Choi et al. 2020; Etherington et al. 2020). Although the Koshihikari draft genome assembled in this study contains numerous contigs, the Nipponbare reference-guided approach compensated for these contigs and led to chromosome-level scaffolding.

Cultivars can be distinguished based on their unique features. The most genomics and genetics studies are conducted to understand the biology behind such variations. The current study attempted to elucidate the causal cultivar-specific genomic regions that contribute to the good EQ of Koshihikari by taking advantage of long-read sequencing. Several genomic regions of Koshihikari showed structural variations compared with Nipponbare. The gapped region in short arm of chromosome 3 was previously identified QTL from Koshihikari and Nipponbare backcross inbred lines (BILs), qOE3, related to the overall eating quality and stickiness of cooked rice. The specific genome structure of the region was observed in detail in this study. A large deletion from 1.2 to 2.33 Mb and insertion of sequences in size of 100 kb was observed in Koshihikari. The consequences of the deletion of some genes like 1,3-beta-glucan synthase component family protein, beta subunit of glyceraldehyde-3-phosphate dehydrogenase, and beta-amylase PCT-BMYI to EQ trait should further be assessed. Takeuchi et al. in 2008 confirmed that the Koshihikari alleles of these major QTLs in short arm of chromosome 3 increased EQ using chromosome segment substitution line containing the aforementioned Koshihikari segment in the Nipponbare background.

One of the gapped regions containing the P5 molecular marker on chromosome 11 was studied closely because the P5 marker was previously developed and used to distinguish the EQ of cooked rice among Koshihikari-derived *japonica* varieties (Ohtsubo et al. 2002; Lestari et al. 2009). The known P5 sequences were aligned to a region which is considerably different from the Nipponbare sequences. Several protein-coding genes were predicted and annotated within a 90-kb candidate region, ranging from 17.55 to 17.64 Mb on the long arm of chromosome 11 (Additional file 1: Table S1). The functions of some of these genes were annotated as hypothetical. Some of these genes contained domains with unknown functions, such as proline-glycine-glycine conserved motif containing (PGG), no apical meristem (NAM), and nucleotide-binding adaptor shared by apoptotic protease-activating factor-1, R proteins, and *Caenorhabditis elegans* death-4 protein (NB-ARC) domains. Little is known about the effects of these domains on the physicochemical properties and EQ of rice. However, some genes within the candidate region were also annotated as related to the cell wall proteins and polysaccharides, based on their sequence similarity with proteins from other species including eukaryotes and prokaryotes. Among the eukaryotes, peptidase A1 domain-containing protein from *Capsicum baccatum*, a putative glycine-rich cell wall protein from *Arabidopsis thaliana*, and the glycine-rich cell wall structural protein 1.8 from *Phaseolus vulgaris* showed high sequence similarity with the predicted genes in the region. Similarly, among the prokaryotes, glycosyltransferase family 4 protein which is involved in catalyzing the lipid cycle reactions in cell wall peptidoglycan biosynthesis (Higashi et al. 1967) from the actinobacterium *Desertihabitans brevis* and lipid A biosynthesis lauroyl acyltransferase from *Methylobrevis pamukkalensis*, a type of bacterial lipopolysaccharides, which are glycolipids that constitute the outer monolayer of the membranes of most Gram-negative bacteria (Preston et al. 1996; Vorachek-Warren et al. 2002) were annotated. This suggests that cell wall-related protein-encoding genes within the P5 molecular marker region might affect the grain quality of rice. In comparison with its parental cultivar, there was no significant difference in various EQ traits of NILs, except for Toyo taste value. Since Toyo taste meter measures the glossiness of cooked grain, this suggests that P5 segment is more associated with other EQ-related factors like structural elements including cell wall components than starch biosynthesis. However, the relationship between the cell wall composition of the endosperm and the EQ of cooked rice needs to be verified.

Koshihikari has been extensively used in breeding program for its superior EQ trait. As a result, numbers of cultivars were generated from Koshihikari or its parents; however, not all the Koshihikari-derived cultivars showed good EQ performance as Koshihikari. In the current study, sequencing reads of most of the high EQ cultivars generated from Koshihikari were aligned to P5 marker region; on the other hand, low EQ cultivars were nearly aligned. In spite of the fact that the cultivars were generated from Koshihikari, the presence/absence of P5 segment was associated with the EQ. We also developed NILs to validate the minor effects of Koshihikari-specific P5 segment to the EQ of Koshihikari rice. The Toyo taste meter measures the glossiness of cooked rice, and the Toyo taste value is known to be highly correlated with the overall eating quality of cooked rice (Saika 1992). In this study, the Toyo taste values of NILs ranged from 51.7 to 57.7, which explained approximately 13.8% of phenotypic variance by the effects of the P5 Koshihikari-specific segments to EQ at most. Although each NIL showed variation in the power of explanation, as expected, it can be explained by environmental factors. Moreover, the application of the results to rice breeding will be feasible by developing additional molecular markers in the candidate region. The results of this study are expected to facilitate the molecular breeding of rice cultivars with good EQ and to help understanding the molecular basis of the quality of rice.

## Materials and methods

### Plant materials and DNA extraction

*Oryza sativa* ssp. *japonica* cv. Koshihikari was used in this study. Koshihikari seeds (accession number IT002752; NAC, RDA, South Korea) were germinated in the dark for 3 days. The seedlings were transplanted into pots and grown in the growth chamber at 24 °C under 16-h light/8-h dark photoperiod and 60% relative humidity. Young leaves of 3-week-old plants were harvested, flash frozen in liquid nitrogen, and stored at − 80 °C. DNA was extracted from the frozen leaf samples according to the high-molecular-weight gDNA protocol of Oxford Nanopore Technologies.

To identify Koshihikari-specific genomic regions associated with its eating quality, Koshihikari was crossed with Samnam, a Korean *japonica* cultivar with poor eating quality, BC3F4 and BC4F4 lines of Samnam//Samnam/Koshihikari were developed (Additional file 2: Fig. S1). Genotyping was performed using the Fluidigm SNP-type genotyping assay following the methods of Seo et al. (2020) using the SNP marker set (Additional file 1: Table S5). All populations were cultivated in the field under the conventional cultivation conditions at the Experimental Farm of Seoul National University.

### Nanopore sequencing

A DNA library for GridION sequencing was prepared using the ligation sequencing kit (SQK-LSK109; Oxford Nanopore Technologies). R9.4.1 flowcells (Oxford Nanopore Technologies) were used for sequencing. Base calling was performed using Guppy (version 4.2.3) with a high accuracy method.

### Illumina sequencing

Libraries with insert size of 500 bp were prepared from the extracted DNA, according to the instructions provided in the Illumina TruSeq DNA Library Preparation Kits v2 Guide. Short-read sequencing was performed on the Illumina MiSeq platform using the MiSeq Reagent Kit v2 (2 $\times$ 250 bp paired-end reads). To improve analysis accuracy, raw data were preprocessed using Trimmomatic (version 0.33) (Bolger et al. 2014), with the following parameters: 3 minimum quality base, 4 sliding window, 20 average quality, and 50 minimum read size.

### De novo assembly

The Koshihikari genome was de novo assembled, as described by Choi et al. (2020) with slight modifications. Adaptor sequences were trimmed using Porechop. The raw sequence reads were corrected using Canu (version 2.1.1) (Koren et al. 2017), and the initial assembly was performed using Flye (version 2.8.2) (Kolmogorov 2019).

The initial assembly was created using the hybrid method to minimize the error rate. The raw Nanopore reads were subjected to four rounds of polishing using Minimap2 (Li 2018) and Racon (version 1.4.20) (Vaser et al. 2017), followed by one round of polishing using Medaka (version 1.2.1) (https://github.com/nanoporetech/medaka). Then Illumina reads were subjected to four rounds of polishing using Pilon (version 1.22) (Walker et al. 2014).

The contigs were scaffolded using RagTag (version 1.0.2) (https://github.com/malonge/RagTag), with a reference-guided scaffolding approach. The Nipponbare IRGSP-1.0 genome was used as a reference.

### Gene prediction and functional annotation

Gene models were annotated using MAKER (version 2.31.11) (Cantarel et al. 2008). Publicly available IRGSP-1.0 transcript and protein data (https://rapdb.dna.affrc.go.jp/) were used as evidence for the pipeline. RepeatModeler (version 1.0.8) (https://github.com/Dfam-consortium/RepeatModeler) identified the repetitive elements, and RepeatMasker (version 4.1.1) (https://www.repeatmasker.org/) detected the repetitive regions. The initial MAKER analysis was followed by ab initio gene prediction, SNAP (Korf 2004), and Augustus (Stanke et al. 2008), generating datasets, which were used to train gene models. Then, a second iteration of MAKER was run. The gene models were visualized using JBrowse 2 (version 1.0.4) (Buels et al. 2016), a customizable genome browser. The functional annotation of a region of interest was performed using BLASTP against UniProt SwissProt. Psi-BLAST was conducted against EggNOG database 4.5 to predict protein sequences with EggNOG annotation descriptions. tRNA and rRNA were predicted using tRNAscan-SE 2.0 and Barrnap 0.9, respectively. The annotations were merged using Annie (version 4bb3980) and GAG (version d80f3fa).

### Validation and structure analysis of the Koshihikari genome

The statistics of the assembled genome were generated using the bbmap stats.sh script of BBTools suite. The gene completion of assembly and annotation was evaluated using BUSCO (version 5.0.0). The Koshihikari draft genome was aligned to the Nipponbare genome using D-GENIES, and synteny was visualized. Genome alignment and structure variation were visualized using Mauve (version 2.4.0) (Darling et al. 2004). The identified structural variations were confirmed using PCR analysis (Additional file 1: Table S4).

### Variant calling and read depth comparison

The cultivars which have Koshihikari in their pedigree were selected and used in whole genome sequencing: Hatsuboshi, Fukunishiki, Homarenishiki, Kitakogane, Sasanishiki, Mineasahi, Kantou 51, Hokuriku 130, Hitomebore, Kinuhikari, Itadaki, Akinishiki, Yumetsukushi, Yumepirika, Sinboi 3, and Hinohikari. DNA library was constructed with insert size of 450–500 bp using TruSeq Nano DNA Library Prep kits (Illumina, San Diego, CA, USA) following the manufacturer’s guide. Prepared libraries were quantified by qPCR according to the Illumina qPCR quantification protocol. The sequencing data of 2 $\times$ 150 bp paired-end reads were generated using Illumina HiSeq X system with a sequencing depth of > 10 × per sample. The adaptors and low-quality bases were removed using Trimmomatic v0.38 (Bolger et al. 2014) using parameters of ILLUMINACLIP:2:30:10 SLIDINGWINDOW:4:15 MINLEN:50. Trimmed reads were aligned to the generated Koshihikari de novo genome as a reference genome using BWA v0.7.17 MEM with default parameters (Li and Durbin 2009). Samtools v1.9 (Li et al. 2009) was used in sorting the aligned reads, and Picard v2.20.2 (http://broadinstitute.github.io/picard/) was used in removing the duplicates. The nucleotide variant calling was performed using HaplotypeCaller function of GATK v.4.1.2 (McKenna et al. 2010), and the heterozygous genotypes were filtered. The aligned reads of each cultivar on the interested region were enumerated using Integrative Genomics Viewer (IGV) v2.11.9 (Thorvaldsdottir et al. 2013) and compared the read depths.

### Assessment of the eating quality of rice

To evaluate the genic region associated with eating quality, the eating quality of Samnam/Koshihikari NILs was assessed over 2 consecutive years. The harvested rice samples were dehulled and polished to 92.2%, and moisture content was measured to 14%. Head rice (33 g) was cooked at 80 °C for 10 min and allowed to sit at room temperature for 5 min. Then, the surface glossiness of cooked rice, which is highly correlated with its palatability, was quantified using the Toyo taste meter (MA-30A; Toyo, Japan). Values of 5 measurements were averaged to obtain one value per sample. Pasting properties were measured using Rapid Visco Analyzer (Newport Scientific, Warriewood, Australia) following the method described in the AACC Method 61 − 02 (American Association of Cereal Chemists 2000); heating cycle (50–95 °C) — hold (95 °C) — cooling cycle (95–50 °C). The measurements were taken in triplicate per sample. Moisture, protein, and amylose content were measured using Near Infrared Grain Tester (AN-820, Kett, Japan) from polished rice.

## Supplementary Information

Below is the link to the electronic supplementary material.

Supplementary file1 (JPEG 267 KB)

Supplementary file2 (JPEG 557 KB)

Supplementary file3 (PDF 1804 KB)

Supplementary file4 (XLSX 55 KB)

## Data Availability

Raw Nanopore sequencing fasta files generated from this study are available in the Sequence Read Archive of NCBI under accession number PRJNA725969. Illumina sequencing fasta files generated from this study can be found under accession number PRJNA725959. The genome assembly of Koshihikari is available under accession number PRJNA725998.
