# Supplementary figures and images for: Sequencing and de novo assembly of the Koshihikari genome and identification of the genomic region related to the eating quality of cooked rice
Source: Mol Breed. 2022 Oct 14;42(10):65. doi: 10.1007/s11032-022-01335-3 (PMC10248671; doi:10.1007/s11032-022-01335-3)

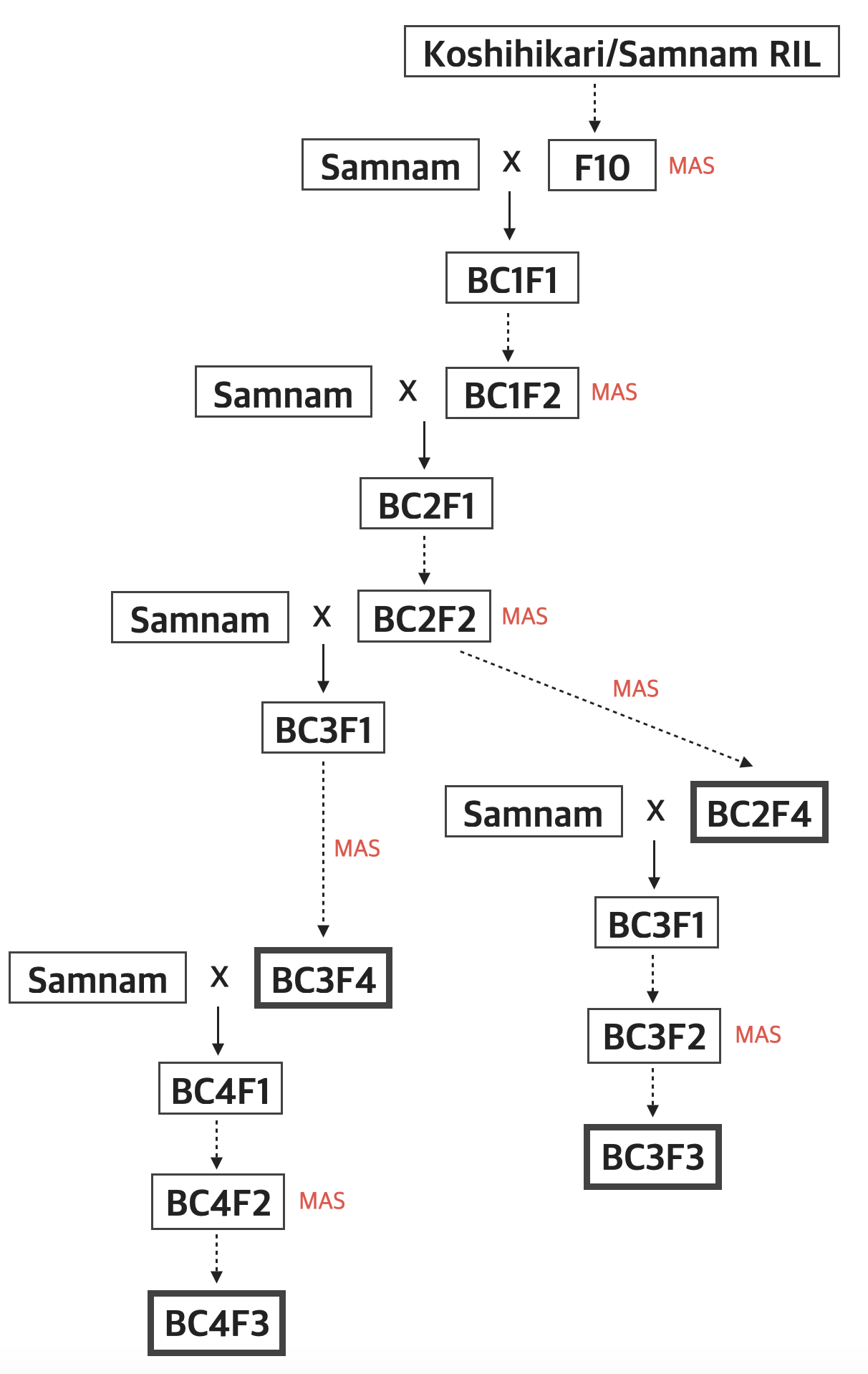

Supplement: Supplementary file 1 — Supplementary file1 (JPEG 267 KB) [file 11032_2022_1335_MOESM1_ESM.jpeg]

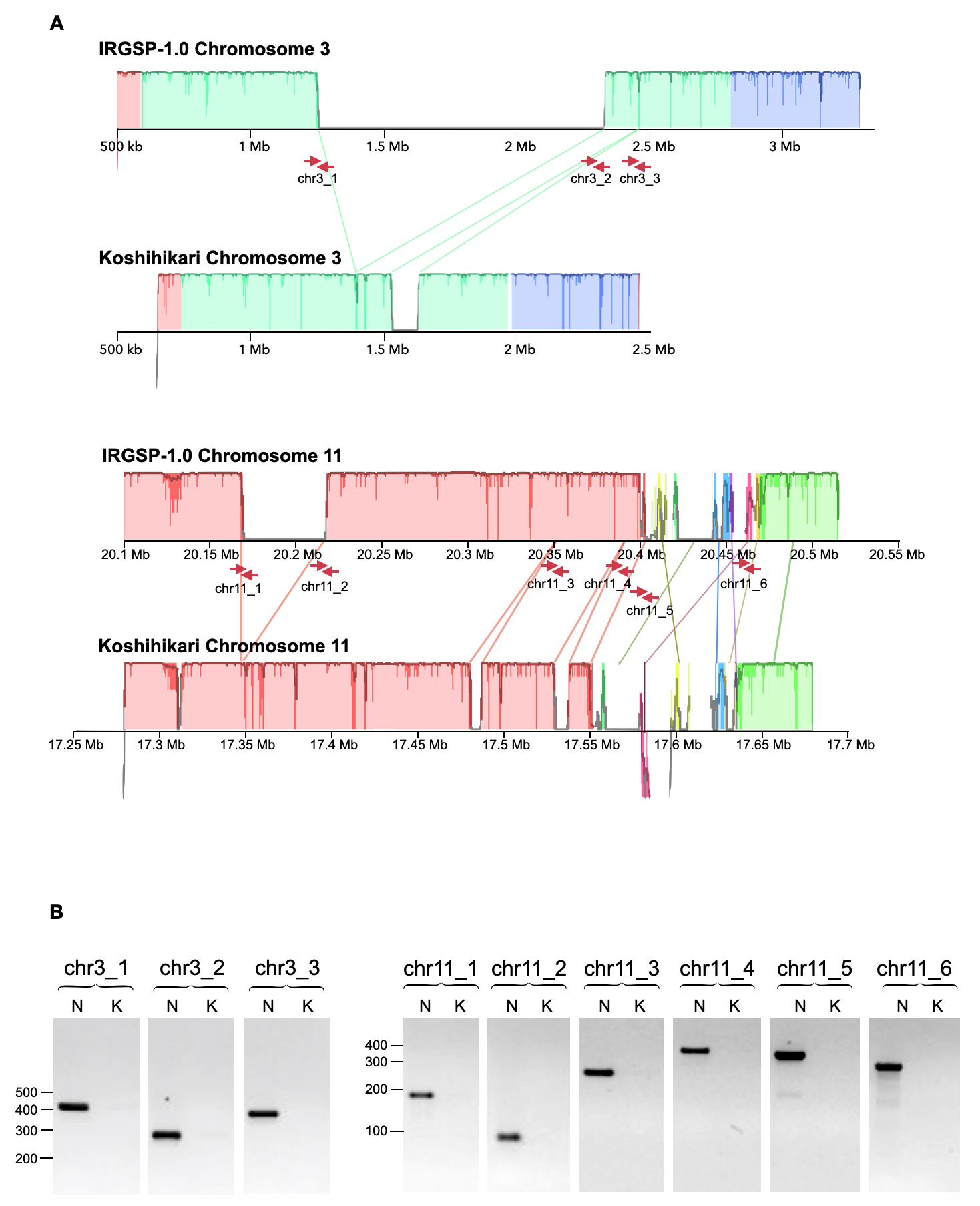

Supplement: Supplementary file 2 — Supplementary file2 (JPEG 557 KB) [file 11032_2022_1335_MOESM2_ESM.jpeg]

**A**

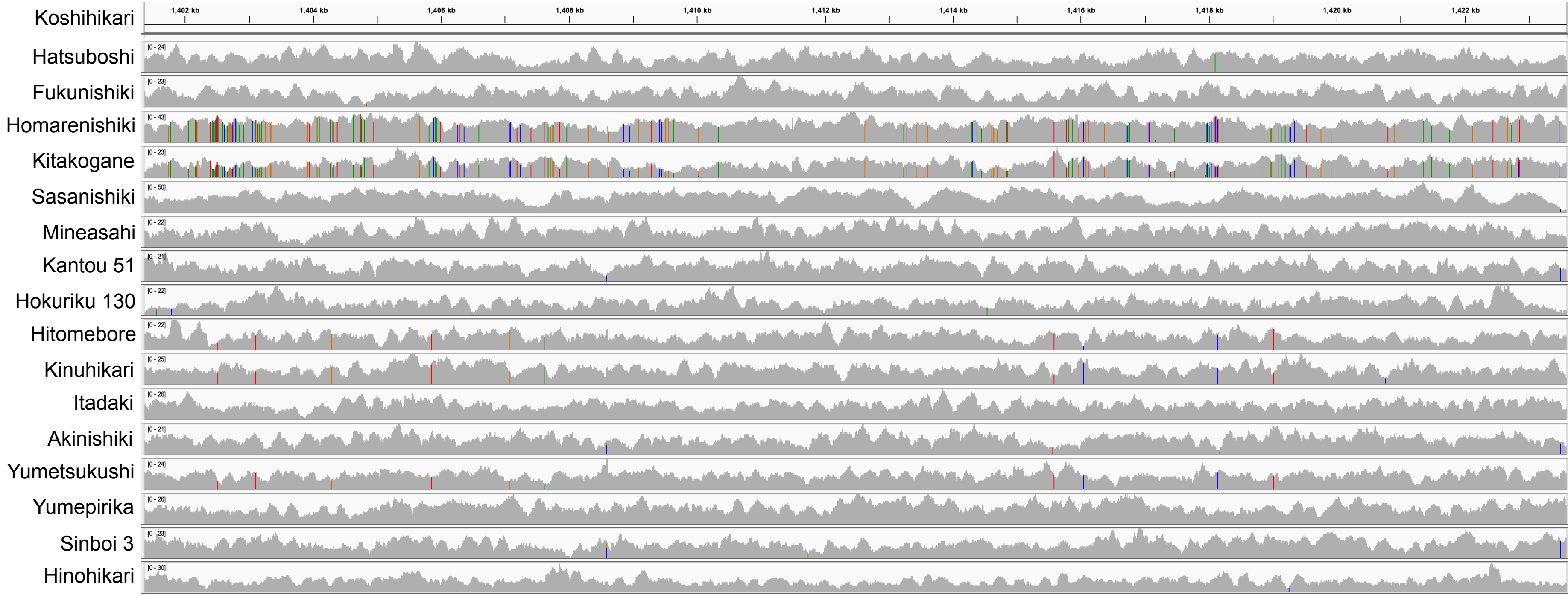

**B**

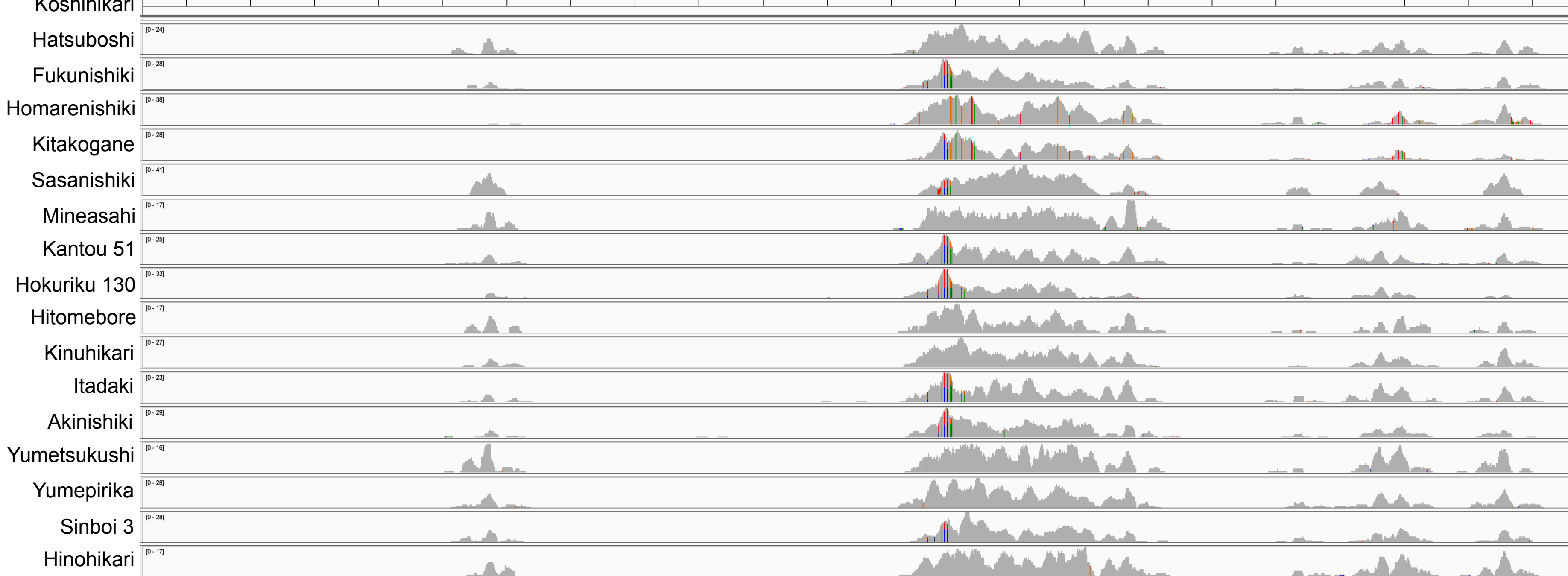

**C**

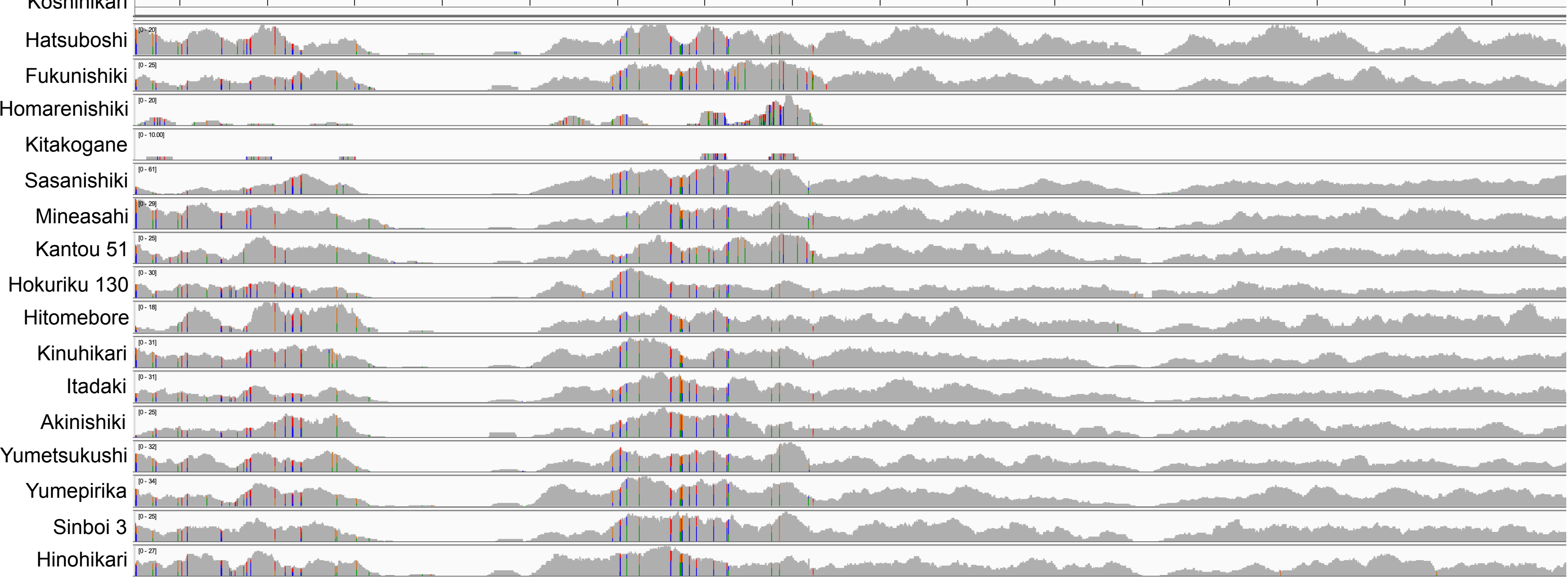

**D**

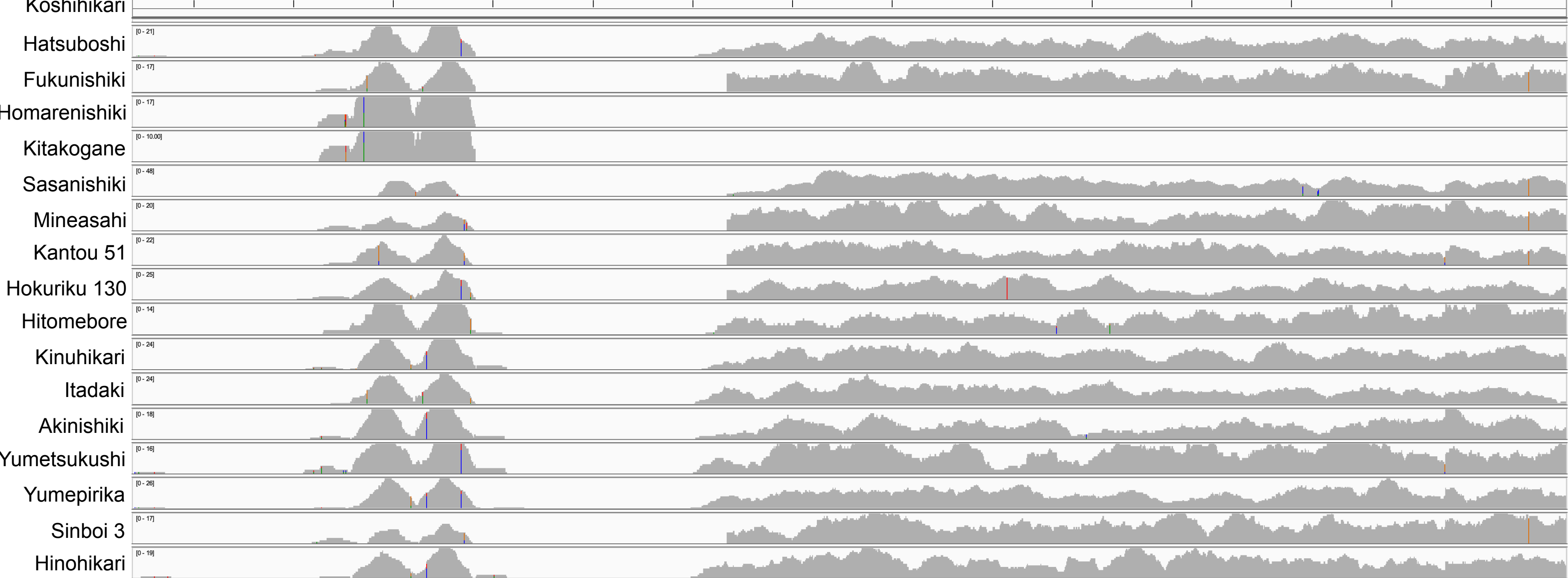

**E**

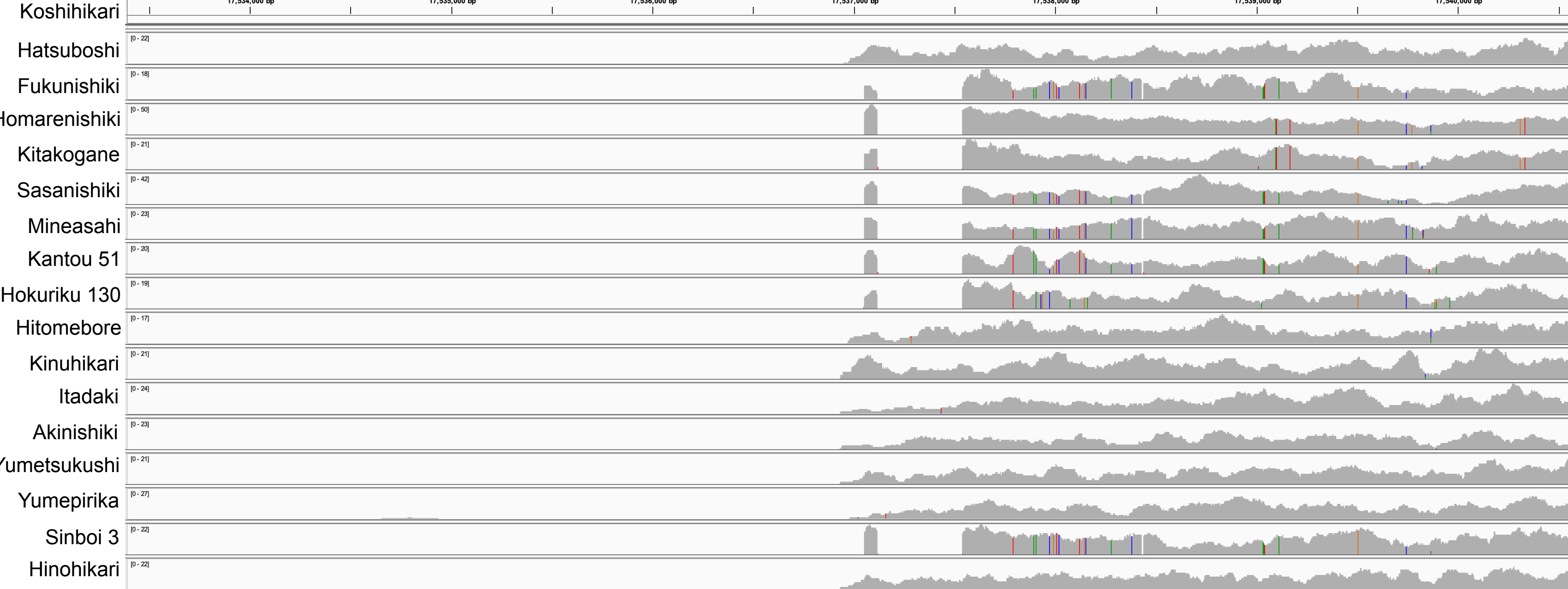

Supplement: Supplementary file 3 — Supplementary file3 (PDF 1804 KB) [file 11032_2022_1335_MOESM3_ESM.pdf]
